# The patient journey with NMOSD: From initial diagnosis to chronic condition

**DOI:** 10.3389/fneur.2022.966428

**Published:** 2022-09-06

**Authors:** Guillermo Delgado-Garcia, Sheryl Lapidus, Rosa Talero, Michael Levy

**Affiliations:** ^1^Department of Clinical Neurosciences, Cumming School of Medicine, University of Calgary, Calgary, AB, Canada; ^2^Centro de Investigacion y Desarrollo en Ciencias de la Salud (CIDICS), Universidad Autonoma de Nuevo Leon, Monterrey, Mexico; ^3^Patient Advocacy, Horizon Therapeutics, Deerfield, IL, United States; ^4^Patient at Neuromyelitis Optica Clinic and Research Laboratory, Massachusetts General Hospital, Boston, MA, United States; ^5^Neuromyelitis Optica Clinic and Research Laboratory, Department of Neurology, Massachusetts General Hospital/Harvard Medical School, Boston, MA, United States

**Keywords:** NMOSD, neuromyelitis optica spectrum disorder, patient journey, diagnosis, patient experience, patient perspectives

## Abstract

**Objective:**

To better understand the patient experience with neuromyelitis optica spectrum disorder (NMOSD) through the course of the illness.

**Background:**

NMOSD is a rare autoimmune disorder that causes recurrent inflammatory attacks of the optic nerve, spinal cord, and brain. Knowledge and awareness of NMOSD in the general medical community are often limited, resulting in potential delays in diagnosis and treatment.

**Design/methods:**

We developed a comprehensive 101-question survey to understand the patient's perspective on their journey from initial presentation to present condition. The survey covered basic demographics, symptoms, medical tests used to reach a diagnosis, and the patient's psychosocial responses to their diagnosis. The survey included questions to determine internal consistency in responses. We shared the survey with members of the Neuromyelitis Optica (NMO) Clinic Facebook group and received responses from 151 patients. All data collected were self-reported and presented as summary statistics.

**Results:**

The majority of survey responses were from patients who were female (83%) and White (76%), Asian (7%), or African American (7%). Initial symptoms of disease included fatigue, pain, stiffness/spasticity, bladder and bowel dysfunction, cognitive/emotional symptoms, and visual disturbances. Initial reactions to NMOSD diagnosis were frequently fear, anxiety, and/or depression. Mean (SD) time to diagnosis was 2.2 (3.2) years. First contact with a medical professional was felt to be not helpful or somewhat helpful for many patients (71%), in part due to uncertain diagnosis and/or treatment. However, once referred to specialists (primarily neurologists), the majority of patients (87%) reported finding a professional who could help. Tests leading to diagnosis included magnetic resonance imaging, lumbar puncture, and blood tests for autoantibodies including aquaporin-4 (AQP4) and myelin oligodendrocyte glycoprotein (MOG). While approximately 30% of patients still felt challenged for a variety of reasons, most patients reported that having a diagnosis and being under the care of a specialist contributed to a comprehensive plan with hope for their future.

**Conclusions:**

The NMOSD patient journey frequently begins with anxiety, fear, and frustration. Finding the right specialist and identifying appropriate screening tests can lead to earlier diagnosis and progression toward better patient outcomes.

## Introduction

Neuromyelitis optica spectrum disorder (NMOSD) is a rare and severe autoimmune disease characterized by inflammation of the optic nerve and spinal cord (1–3). This chronic and potentially debilitating condition is typically marked by multiple relapses that can result in progressive neurologic disabilities, blindness, and even death (1, 3–5). NMOSD has prevalence ranging from 0.5 to 10 per 100,000 in most populations, with considerable global and regional variation (5–8). African Americans are overrepresented in the US patient population (9). A recent survey of patients with NMOSD in North America reported a population who was White (53%), African American (24%), Hispanic (12%), and Asian (9%) (6).

NMOSD was initially considered to be a clinical subtype of multiple sclerosis (MS) as both disorders present with similar symptoms including optic neuritis, myelitis, and demyelination (1, 9–11). NMOSD generally manifests as a series of discrete attacks (1, 9). Relapses occur in 80%-90% of patients, frequently within 1 to 3 years after the initial episode (1, 9). Recovery after an attack often is partial, and the level of disability increases with each relapse, leading to impaired mobility or blindness (1, 9).

Initial symptoms of NMOSD include mild to severe paralysis and ocular pain with loss of vision (1, 9). Other symptoms include intractable hiccups, nausea and vomiting, hearing loss, cranial nerve dysfunctions, sleep abnormalities, narcolepsy, bladder and bowel dysfunction, and acute respiratory failure (1, 4, 12, 13). NMOSD and MS are difficult to distinguish in the early course of disease. The identification of autoantibodies to aquaporin-4 (AQP4-IgG) as highly specific markers of NMOSD has facilitated differential diagnosis (10). Approximately 80% of patients with NMOSD express detectable levels of AQP4-IgG; however, antibody titers by themselves do not seem to be predictive of disease course or outcome (14–16).

The diagnostic odyssey for a patient with NMOSD can be complicated because there is significant variability in clinical presentation and disease course over time (17). NMOSD is frequently misdiagnosed, especially in patients with clinical signs who are seronegative for established biomarkers such as AQP4-IgG and myelin oligodendrocyte glycoprotein autoantibodies (MOG-IgG) (3, 10, 17). Primary care providers and emergency departments, who are often the first points of health care contact, generally have limited or no experience diagnosing and/or treating patients with NMOSD (17–19).

To better understand the challenges and experiences of patients with NMOSD, we explored how patients navigate the early stages of their disease using a survey. The aims of this survey were to identify what patients perceive to be their challenges to diagnosis and treatment and to help health care providers better understand this journey from the patients' point of view.

## Methods

We worked with rareLife Solutions, Inc. to develop a detailed survey to explore the patient's perspective on their initial diagnostic journey from early symptoms to diagnosis and treatment of NMOSD. The survey was shared with members of the Neuroimmunology Clinic (formerly NMO Clinic, Boston, MA, USA) private Facebook group. A pilot survey was administered to a group of 23 volunteers who self-identified as patients. Responses were assessed for completeness, consistency with known baseline values, and demographics for the NMOSD population. The responses obtained from the pilot survey were used to develop a final survey, which was made available in an online format to the full group of patients in the Neuroimmunology Clinic private Facebook group. Survey questions focused on patient population (baseline demographics), signs and symptoms of patients' first clinical events, their initial experiences with the health care system, the diagnostic process, and treatment options. We also focused on the psychological reactions that patients with NMOSD experienced as they were diagnosed with this rare disease. Questions were primarily multiple choice with additional opportunities for patient narratives through inclusion of 6 free-form questions. Survey responses were fielded through SurveyMonkey in a de-identified case report form, and results were collected in September 2020. All data collected were self-reported by the respondents, and the survey could only be completed one time. To participate in the survey, respondents had to agree and grant permission *via* an active response for their data to be used in an aggregated and anonymized manner. Data were anonymized in accordance with General Data Protection Regulation and presented as summary statistics. When narrative responses were reported, any details that could be used to identify respondents were removed.

## Results

Respondents were required to agree to the following statement before they could proceed with the survey: “Please be aware that we will be gathering and processing your responses in total and that while no individual information will be shared with anyone, your responses will be combined and analyzed with all other respondents. Most importantly, your responses will be held in strict confidence. If you are comfortable with that, please continue with the survey, by clicking the button below.”

Patient responses obtained during the pilot survey indicated that patients understood the questions and were actively engaged with the project, as demonstrated by the following: (1) a large percentage of patients answered most, if not all, the questions; (2) patient responses were complete and consistent with known facts about NMOSD; and (3) answers were internally consistent with information provided in response to other related questions in the survey.

Of the 160 volunteers who participated in the final survey, 151 identified themselves as patients, and 9 were advocates and caregivers. Only data from self-identified patients are reported in this article. These data were presented in part as a poster for the 2021 annual meeting of the American Academy of Neurology (20).

### Patient demographics and baseline physical condition

**Figure 1:**
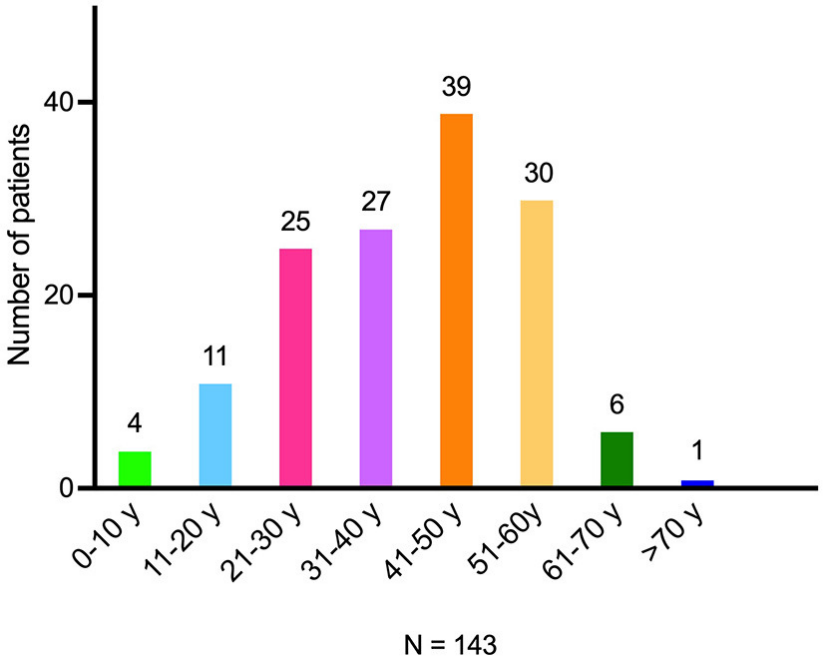
Age at disease onset for NMOSD. NMOSD indicates neuromyelitis optica spectrum disorder.

Respondents to this survey were predominantly female (83%), White (76%), and from the United States (71%) (Table 1), which is representative of the group in general. More than half had completed college or advanced degrees. Median age was 48 (<10 to >70) years (Figure 1) and mean age at disease onset was 40.3 years. Time from diagnosis to this survey was within 4 years for 66/123 (54%) respondents; an additional 40/123 (33%) were diagnosed between 5 and 9 years before this survey, and 15/123 (12%) were diagnosed between 10 and 19 years before this survey. Fifty-two patients reported problems with mobility (requiring a cane, walker, or wheelchair, or being homebound).

**Table 1:** Patient demographics and current level of mobility.

**Characteristic**	**Responses, no. (%)**
Age, median (range), y	48 (<10 to >70)
Sex, *n* = 151	
Female	126 (83%)
Male	18 (12%)
Other/NA	7 (5%)
Race, *n* = 151	
White	115 (76%)
Asian	11 (7%)
African American	10 (7%)
Native American	3 (2%)
Hawaiian/Pacific Islander	2 (1%)
Other/PNtS	10 (7%)
Ethnicity, *n* = 146	
Not Hispanic/Latino	125 (86%)
Hispanic/Latino	12 (8%)
PNtS or NA	9 (6%)
Level of education, *n* = 151	
Advanced degree	37 (24%)
Completed college	50 (33%)
Some college	34 (22%)
Completed high school	22 (15%)
Some high school	4 (3%)
PNtS	4 (3%)
Country/region of residence, *n* = 147	
USA	104 (71%)
Australia	11 (7%)
Canada	9 (6%)
EU	8 (5%)
UK	6 (4%)
Asia	6 (4%)
Other	3 (3%)
Level of mobility at time of survey, *n* = 126	
None	74 (59%)
Need a cane to get around	24 (19%)
Need a walker	11 (9%)
Need a wheelchair	12 (9%)
Confined to home	5 (4%)

NA, no answer; PNtS, prefer not to say.

### Characteristics of first NMOSD attack

**Figure 2:**
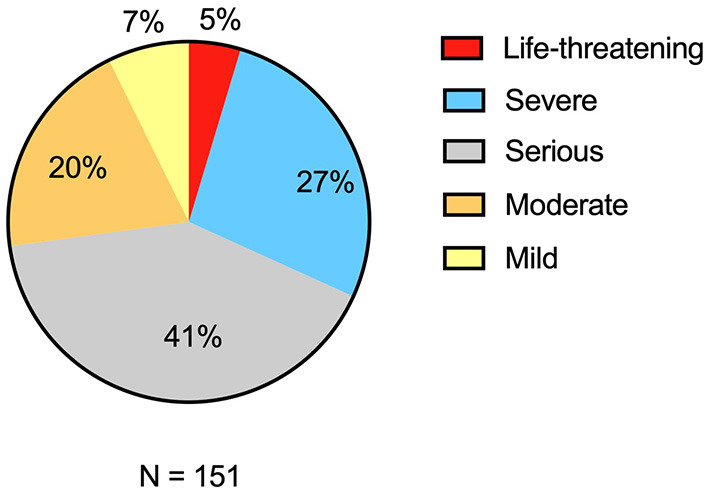
Severity of the first attack of NMOSD. Patients reported severity of first events to range from mild to life-threatening. NMOSD indicates neuromyelitis optica spectrum disorder.

**Figure 3:**
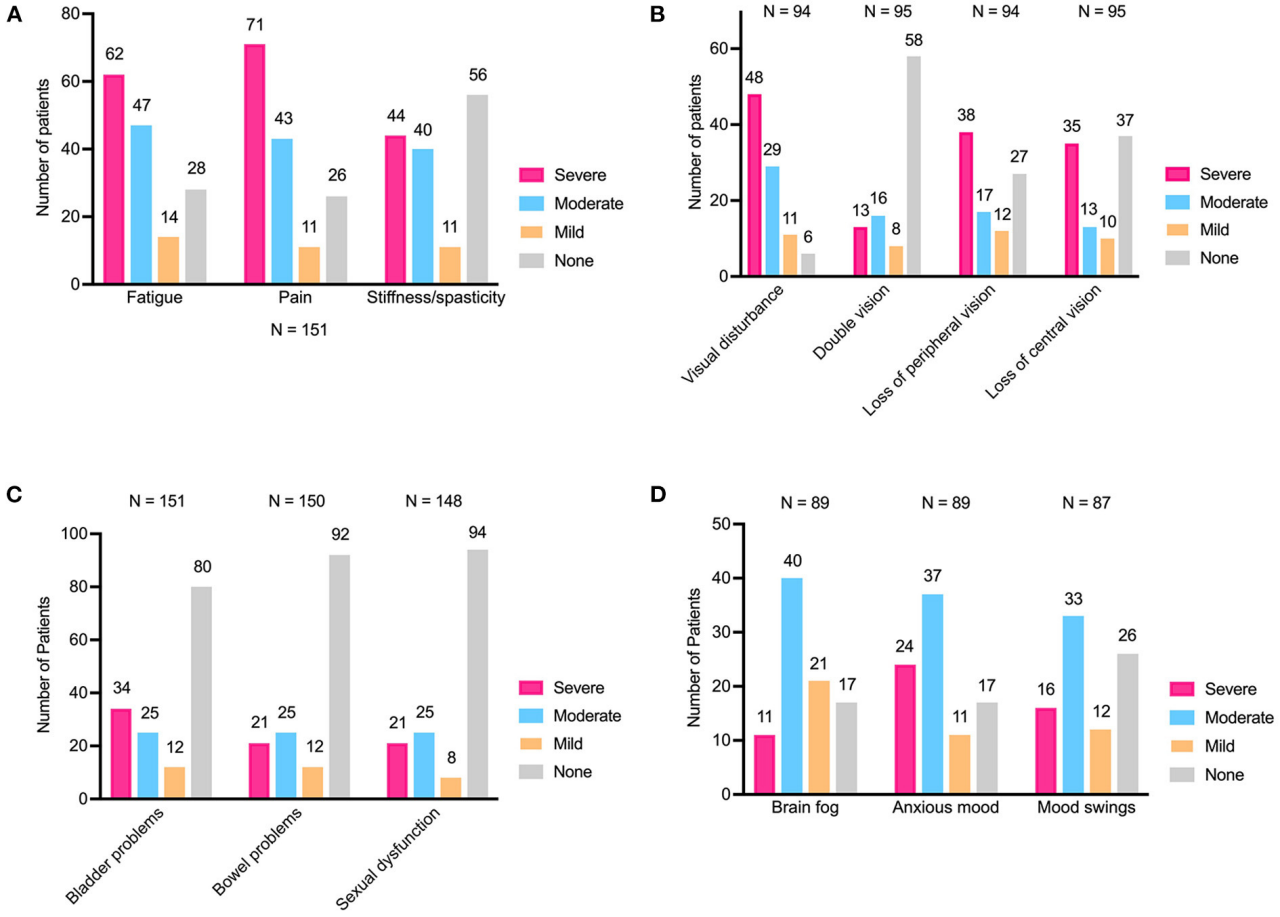
Signs and symptoms encountered during an initial attack of NMOSD. **(A)** Fatigue, pain, and stiffness/spasticity. **(B)** Visual disturbances, double vision, loss of peripheral vision, and loss of central vision. **(C)** Bladder and bowel problems, sexual dysfunction. **(D)** Brain fog, anxious mood, and mood swings. NMOSD indicates neuromyelitis optica spectrum disorder.

In all, 73% (110/151) of patients described their first attack as serious or worse, with 5% (7/151) reporting it as life-threatening (Figure 2). Eightythree percent (125/151) of respondents experienced pain, 81% (123/151) experienced fatigue, and 63% (95/151) experienced stiffness or spasticity (Figure 3A). Of the patients who reported an impact on their vision, 94% (88/94) experienced visual disturbances, 39% (37/95) experienced double vision, 71% (67/94) experienced loss of peripheral vision, and 61% (58/95) experienced loss of central vision (Figure 3B). Patients also reported other physical symptoms including bladder problems 47% (71/151), bowel problems 39% (58/150), and sexual dysfunction 36% (54/148) (Figure 3C). Additionally, cognitive and emotional symptoms were reported by 59% (89/150) of patients and included brain fog, mood swings, and anxiety (Figure 3D).

This survey contained questions that afforded patients the opportunity to write narrative comments about various aspects of their diagnostic journey. Initial attacks of NMOSD were often described as painful and frightening (Supplementary Table 1). One patient described their initial experience as follows: “*Two weeks of severe cold that developed into flu symptoms with headache, weakness, and body aches. I was placed on an antibiotic. The headache worsened and I developed blurred vision and loss of vision in one eye. My antibiotic was changed. Two days later, I developed severe abdominal pain. While in the ER, the weakness progressed to paralysis from the chest down.”*

### First experience with health care system

Patients often described their the initial contact with the health care system using terms such as “scared,” frustrated,” and “bewildered” (Table 2). It was noted that 107 of 151 (71%) patients responded that their first contact with a medical professional was “not helpful” or only “somewhat helpful” in guiding them toward their next steps. Fewer than 10% of patients described their initial contact with a medical provider as “hopeful.” Only 16 of 144 (11%) were diagnosed as having preliminary NMOSD. Initial treatments were prescribed for ~75% of patients and included prednisone/methylprednisolone, gabapentin, baclofen, azathioprine, or rituximab. Other initial treatments offered included antibiotics, pain medications, exercise, and a referral to a psychiatrist. Almost all (148/151) patients provided brief narrative acounts of their initial experiences, coping strategies, and emotional responses to the sudden challenges of their attack (Supplementary Table 2). One patient described their experience as follows: “*Initially, I felt scared and bewildered. No one understood what was going on. There was nothing to help me see better to start school, no treatment suggested to correct my vision[,] and no reason why it was happening. They were just unanswered questions. When the doctors couldn't figure out what was wrong and was happening, they accused me of faking and suggested a psychiatrist to my parents.”*

**Table 2:** Patient first interaction with a health care provider.

**Question**	**Responses, no. (%)**
What type of health care provider did you first visit? *n* = 144	
ER doctor	49 (34%)
Primary care doctor	49 (34%)
Neurologist	26 (18%)
Ophthalmologist	13 (9%)
Other	7 (5%)
What was the first contact with a medical care provider like? How did you feel during, then after the appointment (check all that apply)?^a^ *n* = 150	
Scared	86 (57%)
Frustrated	60 (40%)
Bewildered	56 (37%)
It will go away	40 (27%)
Alone	36 (24%)
Annoyed	30 (20%)
Impatient	20 (13%)
Relieved	15 (10%)
Hopeful	14 (9%)
Grateful	4 (3%)
Was there an initial diagnosis? *n* = 151	
Yes	81 (54%)
No	70 (46%)
What did they attribute your signs and symptoms to (check all that apply)?^a^ *n* = 144	
Preliminary MS	50 (35%)
Stress	28 (19%)
Nonspecific neurologic issue	25 (17%)
Anxiety	20 (14%)
Autoimmune issue	20 (14%)
Preliminary NMOSD	16 (11%)
Other	35 (24%)
Was an initial treatment suggested? *n* = 149	
Yes	113 (76%)
No	36 (24%)
Was the first point of contact with a medical provider helpful in guiding you to what to do next? *n* = 151	
Very helpful	23 (15%)
Yes	21 (14%)
Somewhat helpful	41 (27%)
No	66 (44%)

a Because patients can select more than one option, the total percentage may exceed 100%.

ER, emergency room; MS, multiple sclerosis; NMOSD, neuromyelitis optica spectrum disorder.

### Path toward a diagnosis and treatment

**Figure 4:**
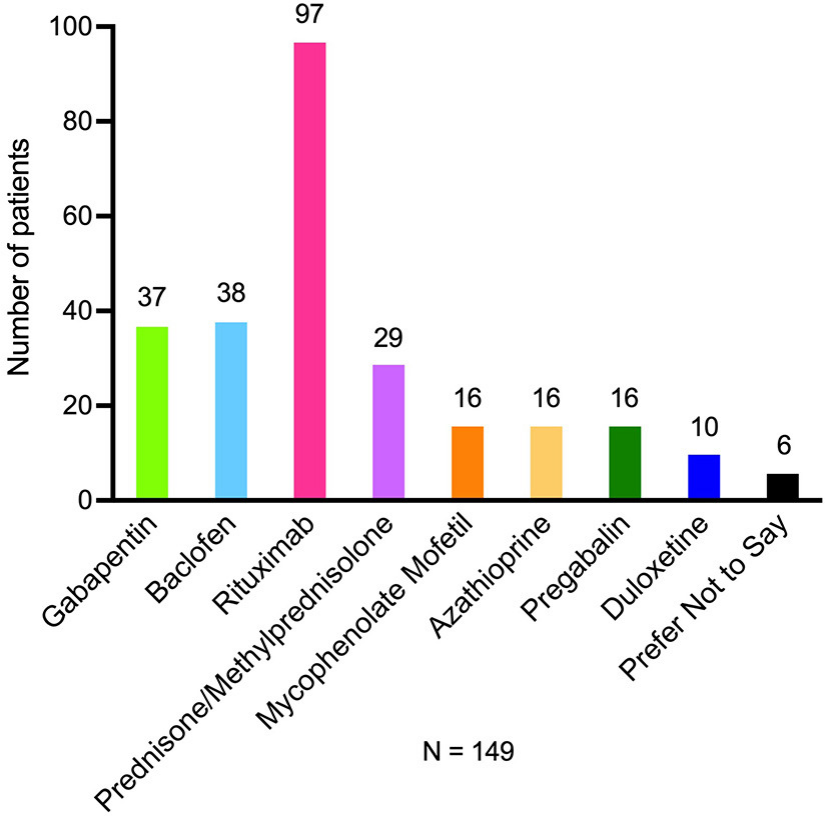
Current medications being used for treatment of NMOSD symptoms. One hundred forty-nine patients reported taking 1.8 medications each (mean) for NMOSD symptoms. NMOSD indicates neuromyelitis optica spectrum disorder.

Time from the first onset of symptoms to a diagnosis of NMOSD ranged from 1 month (20%) to more than 10 years (9%) (Table 3). The mean (SD) time to diagnosis was 2.2 (3.2) years and the median time was 7 months. Many patients subsequently proceeded to seek additional help, and care often transitioned from a general practitioner to a specialist, who was a neurologist for 98% of patients. Over half of patients reported feeling relieved after meeting their NMOSD specialist. Approximately half of patients had to go to a major academic medical center to see their specialist. Travel and time away from home were frequently required for patients to see their specialist, but travel was rarely international. Clinical and laboratory tests used to confirm NMOSD included physical examination, blood tests, magnetic resonance imaging, and lumbar puncture (Table 4). After the first series of tests, 99 of 151 (66%) of patients had to undergo further extensive tests which often included additional imaging and radiology. Seventy-six (69%) of the 110 patients who reported being tested for AQP4-IgG; had a positive response, and 18 (32%) of the 56 patients who reported being tested for MOG-IgG had antibodies. Approximately two-thirds of patients reported that they were provided with the appropriate information to help them understand their diagnosis of NMOSD. Patients reported that they were taking a mean of 1.8 medications for NMOSD, and almost two-thirds of them were taking rituximab (Figure 4). Approximately half of the respondents had received at least one plasmapheresis treatment.

**Table 3:** Patient transition from a general practioner to a specialist.

**Question**	**Responses, no. (%)**
Time from symptom onset to NMOSD diagnosis, *n* = 150	
• 1 month• 2 months• 3 months• 4 months• 5 months• 6-11 months• 1 year• 2–5 years• 6–10 years• >10 years	30 (20%) 10 (6%) 11 (7%) 13 (9%) 6 (4%) 13 (9%) 11 (7%) 31 (21%) 12 (8%) 13 (9%)
What type of specialist did you see (check all that apply)?^a^ *n* = 136	
• Neurologist• Immunologist• Psychiatrist• Other	133 (98%) 11 (8%) 6 (4%) 5 (4%)
To see this specialist, did you have to go to a major academic medical center? *n* = 134	
• Yes• No	76 (57%) 58 (43%)
Did this require significant travel and time away from home? *n* = 76	
• Yes• No	44 (58%) 32 (42%)
Was the travel international? *n* = 75	
• Yes• No	4 (5%) 71 (95%)
Did it present any language barriers? *n* = 4	
• Yes• No	2 (50%) 2 (50%)

a Because patients can select more than one option, the total percentage may exceed 100%.

NMOSD, neuromyelitis optica spectrum disorder.

**Table 4:** Medical procedures/tests informing the diagnosis of NMOSD.

**Question**	**Responses, no. (%)**
What initial medical testing did you receive as part of your first visit (check all that apply)?^a^ *n* = 128	
• Blood tests• MRI• Physical exam• Spinal tap• X-rays• Other	114 (89%) 112 (88%) 97 (76%) 88 (69%) 37 (29%) 3 (2%)
Did you then undergo more extensive and invasive medical tests after the first series? *n* = 151	
• Yes• No	99 (66%) 52 (34%)
If yes, what more extensive and invasive tests were performed (check all that apply)?^a^ *n* = 99	
• MRI• Spinal tap• Other imaging• Radiology• Other	91 (92%) 61 (62%) 45 (45%) 34 (34%) 9 (9%)
Did you undergo more extensive blood tests, including detailed screens for a range of autoantibodies? *n* = 151	
• Yes• No• Not sure	126 (83%) 12 (8%) 13 (9%)
Which autoantibodies were you positive for (check all that apply)?^a^ *n* = 122	
• AQP-4• MOG• Not sure• None• Other	76 (62%) 18 (15%) 24 (20%) 11 (9%) 3 (2%)
As the patient, were you provided with the appropriate information to better understand your diagnosis of NMOSD? *n* = 149	
• Yes• No	92 (62%) 57 (38%)
Once you received a diagnosis of NMOSD, did you wonder about how your disease would progress? *n* = 151	
• Yes• No	144 (95%) 7 (5%)
What questions did you have (check all that apply)?^a^ *n* = 151	
• What will my future look like?• Will I get better?• Will I get back to feeling normal?• If not, what will be my new normal be like?	135 (89%) 118 (78%) 120 (79%) 110 (73%)

a Because patients can select more than one option, the total percentage may exceed 100%.

AQP-4, aquaporin-4; MOG, myelin oligodendrocyte glycoprotein; MRI, magnetic resonance imaging; NMOSD, neuromyelitis optica spectrum disorder.

After meeting with an NMOSD specialist, 132 of 151 (87%) patients reported that they felt they had access to a professional who could guide them with treatment decisions (Table 5). In all, 106 of 150 (71%) respondents stated that they understood and could take advantage of their best treatment options, and 105 of 150 (70%) had a comprehensive care and recovery plan in place. After receiving their diagnosis and beginning to work with an NMOSD specialist, the majority of patients reported feeling relieved; however, others felt unhappy or lost. Upon diagnosis, patients had to confront their new reality of having NMOSD (Supplementary Table 3). “*It was hard being diagnosed. I was a month and a half away from getting married. I had always been healthy up until I wasn't. I had no real medical history. I was so scared of what the future would hold. Would I be blind? Would I be in a wheelchair? Would I be able to have children? Would I be dead in 5 years?”*

**Table 5:** Identification of treatment options after a definitive diagnosis.

**Question**	**Responses, no. (%)**
How did you feel after meeting your NMOSD specialist? *n* = 111	
• Relieved• Unhappy and lost• Other	86 (77%) 16 (14%) 9 (8%)
Do you feel like a comprehensive care and recovery plan is in place? *n* = 150	
• Yes• No	105 (70%) 45 (30%)
Based on the details of my specific situation, do I feel that I understand and can take advantage of my best options? *n* = 150	
• Yes• No• Not sure	106 (71%) 14 (9%) 30 (20%)
Do you feel like you know, and have access to, the professional who will guide/help you in making these decisions? *n* = 151	
• Yes• No	132 (87%) 19 (13%)
If not, why do you feel that you do not know and/or have access to this professional? [Free-form answer] *n* = 17	NMOSD specialist is too far away No expert doctor Months to get an appointment Diagnostic issues Public health care limitations

NMOSD, neuromyelitis optica spectrum disorder.

After a period of mourning their old lives and accepting the permanent losses, patients frequently began adjusting to a “new normal.” When asked whether patients felt confident that they can now “live your best life,” the responses were more positive than negative, although many patients still struggle with a life of limitations (Supplementary Table 4). “*I'm adjusting to my new normal. But I feel like every time something new goes numb, or something doesn't feel right, I have to wonder if it's an [NMOSD] attack. So, dealing with the unknown is a fear I live with every day.”*

## Discussion

Our survey provides information that describes the symptoms of the initial attack of NMOSD and patients' reactions to this experience while navigating the health care system to the point where a correct diagnosis was obtained. This survey is the first, to the best of our knowledge, that focuses on the patient's initial NMOSD attack and provides a substantial opportunity for patients to provide narrative responses regarding their feelings and reactions to their experience. Our patient population had essentially the same characteristics as those in other surveys of patients with NMOSD, suggesting that they are representative of the NMOSD populations who participate in surveys (17, 21–24). Unlike in previous surveys that used standardized assessment instruments, we intentionally designed ours to allow patients to express their feelings in a free form. Despite the subjective nature of our survey, our results were very similar to those from surveys that utilized standardized tools with the additional important benefit that we were able to obtain very personal insights into patients' feelings and psychological state as they navigated their path through diagnosis and treatment (17, 21–24). In future surveys, it would be of interest to go even deeper into patient experiences to explore issues such as how regional differences affect their journey and how NMOSD has affected their ability to work and interact in society.

We believe that the NMO Clinic private Facebook community was highly motivated to share their journeys, as indicated by the number of patients completing the long and detailed survey. A large percentage of patients provided thoughtful narrative answers where appropriate. We believe adding questions that could elicit narrative responses enabled the patients to delve more deeply into questions about their quality of life and emotional experiences. For example, 148 out of 151 patients (98%) responded to questions about their coping strategies and emotional reactions to their diagnostic experience.

Patients' descriptions of their first attack of NMOSD and their contact with medical professionals clearly demonstrate how distressing the process can be. Patients describe fear, frustration, and disappointment. Patients describe how they were often confronted with sudden, distressing, and painful attacks of NMOSD with relatively little support or understanding from the medical community, especially emergency departments, primary care physicians, and neurologists, due to lack of knowledge of NMOSD (19). Increased understanding of NMOSD by physicians can help preserve vision and avoid permanent disability as well as help patients transition more efficiently to the right specialists (19). Finding the right specialist and identifying appropriate screening tests can lead to an earlier correct diagnosis and faster progress toward better treatment and outcomes (10, 19).

Understanding the patient journey can yield important insights that could have a beneficial impact on patient care. This Facebook group and other social media networks like PatientsLikeMe provide access to many patients who have NMOSD and should be utilized to expand awareness to a broader patient and physician population (23). Patient responses to this survey provided detailed insights into the challenges that they encountered as they tried to find the best path forward in their new life. Utilizing patient narratives in publications can help clinicians empathize with the experiences that are often so frightening and disturbing to their patients (25–27). We believe that adding narrative questions within this survey may have allowed respondents to more freely express their feelings, helped them believe that they were being heard, and helped them to be more engaged in this survey.

The goal of this survey was to gather information on current patient experiences to help improve the patient journey in the future. Based on the responses of several patients, it appears that more education for the medical community could help raise awareness of NMOSD and could help physicians correctly diagnose the disease as early as possible. Many patients spend a long time with a misdiagnosis, which not only aggravates their medical condition but also subjects them to great emotional and financial hardship. An early and correct diagnosis with immediate treatment would be of great value in controlling the damage caused by NMOSD.

### Limitations

As this survey was designed to elicit self-reported responses, individual experiences can be very subjective and less likely to provide quantitative data about specifics of NMOSD. Respondents may have very different perceptions of what “mild” or “serious” means with respect to disease or symptom severity. Moreover, they were often asked subjective questions about their feelings and perceptions. There are also challenges validating a patient's identity and diagnosis through a social media platform. There was no restriction on members of the group sharing the survey link externally, and no validation process was used to confirm that the respondents were in fact patients with NMOSD.

Data collected in this survey came primarily from patients in the United States (71%). Results cannot necessarily be generalized and may differ between regions and health care systems. A potential limitation of this study is that respondents were those who volunteered to complete this online survey. Therefore, individuals without access to the internet or who were unable to see or have the strength to participate were unlikely to complete the survey unless they had a friend or family member complete it with them. Although we queried the status of each respondent (patient, caregiver, or advocate), we did not expressly ask whether respondents were being aided by another person. No person was purposely excluded from the survey, and we did not specifically ask whether respondents were capable of completing the survey unaided.

## Conclusions

Patients with NMOSD face a diagnostic journey that frequently begins with fear, confusion, and frustration. Initial contact with the medical community in the form of emergency departments or primary care physicians can often lead to misdiagnosis due to lack of knowledge about this rare disease. The survey indicates that when given the opportunity, patients are willing to share their experiences in their own words. As patients connect with specialists who provide the correct diagnosis of NMOSD and a treatment plan is developed, patients frequently experience hope for an improved “new normal.”

## Data availability statement

The raw data supporting the conclusions of this article will be made available on reasonable request.

## Ethics statement

Ethical review and approval was not required for the study on human participants in accordance with the local legislation and institutional requirements. The patients/participants provided their written informed consent to participate in this study.

## Author contributions

GD-G and ML were involved in the development of the survey. All authors were involved in the interpretation of the results.

## Funding

Medical writing services were supported by Horizon Therapeutics.

## Conflict of interest

GD-G has received research support from the Consejo Nacional de Ciencia y Tecnologia, Mexico. ML received consulting fees from Alexion (now Alexion, AstraZeneca Rare Disease), Genentech/Roche/Chugai, Sanofi, UCB Pharmaceuticals, and Viela Bio (since acquired by Horizon Therapeutics). SL is an employee of Horizon Therapeutics and holds stock in the company. RT is a patient participating in a clinical trial of ravulizumab sponsored by Alexion, AstraZeneca Rare Disease. The authors declare that this study received funding from Horizon Therapeutics for the preparation and submission of the manuscript. The funder was involved in the review of this article and the decision to submit it for publication.

## Publisher's note

All claims expressed in this article are solely those of the authors and do not necessarily represent those of their affiliated organizations, or those of the publisher, the editors and the reviewers. Any product that may be evaluated in this article, or claim that may be made by its manufacturer, is not guaranteed or endorsed by the publisher.
