# Supplementary material for: The patient journey with NMOSD: From initial diagnosis to chronic condition
Source: Front Neurol. 2022 Sep 6;13:966428. doi: 10.3389/fneur.2022.966428 (PMC9488131; doi:10.3389/fneur.2022.966428)
Supplement: Supplementary file 1 [file Data_Sheet_1.docx]

**Supplementary Material**

**Supplementary Table 1.** Sample of Patient Descriptions of Initial NMOSD Attack

| Two weeks of severe cold that developed into flu symptoms with headache, weakness, and body aches. I was placed on an antibiotic. The headache worsened and I developed blurred vision and loss of vision in one eye. My antibiotic was changed. Two days later, I developed severe abdominal pain. While in the ER, the weakness progressed to paralysis from the chest down. |
| --- |
| Headache and severe pain from back to bottom and in less than 48 hours complete loss of vision and numbness and difficulty of control[ling] the bowel. |
| Unable to see, speak, or move right leg. |
| My first attack consisted of double vision, uncontrollable hiccups, numbness[,] and tingling in arms and legs, vomiting for a three-month span, headaches, and sensation of burning on face. All of these seem to be very severe symptoms [and] it caused immense pain and immobility. |
| Couldn’t walk and my speech was severely affected. My coordination was also affected. My family had to bathe and feed me. |
| Numb from chest to foot[,] left leg[,] and chest down right side. Loss of use of both legs. Tightness in both feet. Confusing sensations in bowels and bladder. Strapping around stomach and chest like wearing laced up corset. Weakness and fatigue. Difficulty focusing. I had been experiencing neurological electric shock like pains for a few years before this. |
| Pain under ribs, temporal headache, increasing weakness, then sudden lack of control of the body. Spasms, then vision problems developing over a few days. |
| Headache for multiple days and eventually a veil over my left eye, slowly diming more each day over the span of 3. Severity high, I lost a lot of balance and wasn’t able to think right. In excruciating head and eye pain. |
| I experienced severe nausea, vomiting[,] and flu-like symptoms. It was very severe. I lost 30 lb. in less than 3 months. Towards the end of the 3-month period I developed bilateral optic neuritis. |
| Throbbing headache at the front and back of my head. Loss of peripheral vision [and] then 10 days later completely black and grey vision. No light perception/strong sensitivity to the light[. I] had to be in darkness and could not see any colors at all or any faces, fingers[,] and letters. Extremely blind. |
| I was extremely concerned because I didn’t know what was wrong with me. Emotionally I was a wreck. I kept taking care of my family and praying. Praying that a doctor would see me face to face and hoping that it wasn’t MS. |
| I was scared and aggravated trying to find answers. I felt hopeless and isolated. Tried to drink away the hopeless feelings as I continued to advocate for myself. |

Abbreviations: ER, emergency room; MS, multiple sclerosis; NMOSD, neuromyelitis optica spectrum disorder.

**Supplementary Table 2.** Samples of Patient Reactions to Contact With the Health Care System and Subsequent Specialists

| Initially, I felt scared and bewildered. No one understood what was going on. There was nothing to help me see better to start school, no treatment suggested to correct my vision[,] and no reason why it was happening. They were just unanswered questions. When the doctors couldn’t figure out what was wrong and was happening, they accused me of faking and suggested a psychiatrist to my parents. |
| --- |
| I was very scared because no one had any answers for me on what was going on. Life as I knew it was in chaos. I would literally sit by the toilet in the middle of the night crying. I just wanted answers, and no one had any for me. |
| First [I] went to a walk-in clinic before my vision was completely gone in my left eye[. T]hey sent me to an eye doctor and after the eye test came back fine, they ordered an MRI of [the] optic nerve[,] confirming optic neuritis. I was then treated with a high dose of [IV] steroids which stopped the attack and pain, but didn’t necessarily help with regaining eyesight[,] although some was restored over a longer period of time. |
| Went [to the] walk-in clinic [for] ambulatory care once [and the] ER twice before being rushed to [the] trauma unit. Initial DX was meningitis. |
| Over a period of about 2 months (after a severe upper respiratory infection), I experienced vomiting and fatigue. That was followed by numbness in my toes, inability to empty my bladder, and the loss of vision in one eye. All symptoms were checked out by the appropriate specialists, none of which realized that they were all connected. The loss of vision was correctly treated with IVSM, but diagnosed as MS after a negative NMO IgG test.  As the IVSM for the optic neuritis took effect, my vomiting stopped, and my bladder slowly normalized. I was eventually put on a prednisone taper which stretched across 2 months. As the taper ended I had sharp pains in the center of my back. A few days later I felt as if my buttocks were vibrating if I sat for any period of time, and a few days after that, I began experiencing weakness in my legs and within 48 hours I was paralyzed from armpit level to my toes. And I again lost the ability to empty my bladder. The paralysis caused an inability to cough or blow my nose, and my right arm and hand became weak and shaky. I never lost sensation in my legs and feet, but I did have altered sensations and complete inability to move my legs as well as the inability to sit up unassisted. |
| Neuro gave me [venlafaxine] thinking my symptoms [of] pain and numbing, banding, etc. [were] anxiety.  I don’t have an answer except my desire to survive is strong. I live in fear everyday feeling I’m going to drop at any moment. |
| I was scared. I had no clue what was happening to my body. I went to my PCP and then to the ED and the doctor at the ED treated me like I just wanted drugs. He had no answers and sent me home. I finally went to a larger hospital ED and was admitted for 23 days until the vomiting finally stopped. |
| I did not know what was happening to me. [I w]ent [to the] doctor several times but did not get any help. I felt confused and desperate, and it was really hard to get through my work shifts. |

Abbreviations: DX, diagnosis; ED, emergency department; ER, emergency room; IV, intravenous; IVSM, intravenous solumedrol; MRI, magnetic resonance imaging; MS, multiple sclerosis; NMO IgG, aquaporin-4 antibodies (AQP4-IgG); PCP, primary care provider.

**Supplementary Table 3.** Samples of Patient Experiences and Reactions After NMOSD Diagnosis

| It was hard being diagnosed. I was a month and a half away from getting married. I had always been healthy up until I wasn’t. I had no real medical history. I was so scared of what the future would hold. Would I be blind? Would I be in a wheelchair? Would I be able to have children? Would I be dead in 5 years? |
| --- |
| Was evacuated by air ambulance from [foreign country] to [a specialty center in the US]. Was having seizure-like events, concern I would die in [foreign country]. [A specialty center in the US] diagnosed [NMOSD] [based] on spinal fluid. Treatment by intense IV prednisone, later rituximab. Months of emotional counseling to deal with permanent losses. Took me a couple of years. |
| After 10 years of no diagnosis, it was comforting to have answers. I then did as much research as possible so I could understand the disease and what to possibly expect and be aware of. |
| The ON affected my left eye only, so I was able to continue to function in everyday life. However, my life of sports changed because of the loss of depth perception. Psychologically, having [NMOSD] was definitely a negative change; however, with the help with family and my specialists at [a specialty center in the US]. I was able to look on the positive side of this disease. Also, having the opportunity to go to the [NMOSD] workshops put on Guthy-Jackson Foundation definitely allowed me to put the illness in perspective. Listening to others and the professionals studying [NMOSD] was a very positive influence. |
| My [NMOSD] doc and his team responds to my health questions regarding [NMOSD] very quickly online. If needed, he requires an appointment with his team. He stays on top of all needed labs and tests. He keeps me informed of updates regarding [NMOSD] during my appointment with him. |
| I am lucky to have a neurologist who has treated other NMO patients successfully, made a quick diagnosis, and started the correct treatment immediately. She recommended one of the top rehab hospitals for inpatient treatment, and I felt extremely well prepared when I was discharged two months later. |
| Initially really worried without any diagnosis, families really supported me, moved from one doctor to another to really identif[y] what [was] going on, at [the] end God [lit] up my life with [the NMOSD] diagnosis, whole journey was like it will [be] over one day and I will be fit again, till today I think [a] cure will be soon and I will be fit again. |

Abbreviations: IV, intravenous; NMO, neuromyelitis optica; NMOSD, neuromyelitis optica spectrum disorder; ON, optic neuritis.

**Supplementary Table 4.** Sample of Patients’ Reactions to Adjusting to a “New Normal” and Living Their “Best Life” With NMOSD

| I am not comfortable knowing that any day I could relapse. I just try to live every day the best and fullest without thinking about what might happen. |
| --- |
| I am doing the best I can. I used to feel like there was a sniper over my shoulder ready to take a shot, but I don’t really care anymore. I can live until I’m old or die tomorrow. That is out of my hands, so I live for now. I get jealous of my former self and seeing people doing energetic things, but this is my new normal and I am adjusting as well as I can. :) |
| Yes, I adapt to my new normal and do my best. |
| Not sure what expectations will be realistic but hoping for the best. |
| No, I don’t think this is my best life. Just making do with my new normal and adjusting accordingly. |
| I’ve learned how to accept my new normal. There are days I get extremely frustrat[ed] because I can’t do a lot of what I used to do. I just have to accept that this is how my life is now and adapt to new ways to do things. |
| I feel I’ve adapted to a new normal with the permanent blind spot that I have. With the challenges that come with having relapsing NMO, yeah, I feel I should be able to live my best life. |
| Yes. With the help of my neurologist, I am getting the best treatment and living a “normal” life. He saved my vision. |
| Yes, for the most part I am living my best life, I have recovered from my attacks fairly well. Although I do live with the constant worry of, what is next? How long will I have healthy/“normal”? |
| Not the best [life]. Definitely not what I thought it would be. The best as far as it can get, I guess. I won't be running a marathon, but I’d be happy if I could walk in a 5 km again. |
| It took a while, but I’ve got a great group of doctors ... in that aspect I feel comfortable, but I’m worried about my health insurance and how I’m going to support myself because I’m not working. I’m trying to get onto permanent disability but it’s a very difficult process. People like me could use help with it. |
| Not my best life but [I’ve] learnt to grieve the old life and accept the new. |
| I've been doing so [living my best life] for the past 11 years, since my diagnosis. |
| Maybe lol. I will try. |

Abbreviations: NMO, neuromyelitis optica; NMOSD, neuromyelitis optica spectrum disorder; ON, optic neuritis.
